# Breaking through the noise: how to unveil the cognitive impact of long COVID on pre-existing conditions with executive dysfunctions?

**DOI:** 10.3389/fpsyt.2024.1390214

**Published:** 2024-05-23

**Authors:** Caroline Jose

**Affiliations:** ^1^ Centre de médecine de précision, Centre hospitalier universitaire Georges-L.-Dumont, Réseau de santé Vitalité, Moncton, NB, Canada; ^2^ Département de médecine de famille et de médecine d’urgence, Faculté des Sciences de la santé, Université de Sherbrooke, Sherbrooke, QC, Canada; ^3^ Département de chimie et biochime, Faculté des sciences, Université de Moncton, Moncton, NB, Canada

**Keywords:** long COVID, pre-existing cognitive impairment, cognitive sequelae, neurodevelopmental conditions, executive dysfunctions, Patient-oriented Research

I am a Canadian patient-oriented researcher working with patient partners with cognitive disorders [notably, autism, learning disorders, and attention deficit/hyperactivity disorders (ADHD)]. During work sessions together, I realized that their experience with the coronavirus disease 2019 (COVID-19) infection felt very different than mine. Those who had been infected explained to the group that the virus had magnified their daily struggles and sometimes still does 3 years later. Symptoms like insomnia, short-term memory issues, sensory overload, and concentration issues, with which they have been dealing for decades, became out of control and very distressing.

Knowing that the long-term sequelae of COVID-19 infection impact similar cognitive processes in a third of infected patients months after the acute phase (1), asking if patients with pre-existing cognitive disorders may be at increased risk of further cognitive impairment following COVID-19 is a fair question. For my fellow partners with neurodevelopmental disabilities, the question is even essential, as, if backed with evidence, it could enable access to long COVID care.

Systematic reviews and meta-analyses have become increasingly important in healthcare settings and are the main starting point for developing clinical practice guidelines. However, regarding the topic of the long-term interplay between neurodevelopmental disorders and the neurocognitive involvement of COVID-19, they might not be of great help. In two authoritative JAMA meta-analyses on long COVID prevalence and risk factors (1, 2), cognitive disorders are not once mentioned. This is not surprising since the individual studies they included were seldomly reporting on neurological or psychiatric disorders in the clinical characterization of their samples. The lack of reporting on those comorbidities is not the only barrier to better understand the potential association between cognitive disorders and long COVID cognitive sequelae. Most studies analyzed in those two meta-analyses excluded directly or indirectly the participation of people with cognitive impairments. Despite legal protections and policy directives for vulnerable groups, excluding patients with cognitive impairments who are unable to provide consent is common practice in clinical research (3). Long COVID-related randomized clinical trials (RCTs) are no exception. Seven clinical trials published in 2022 and 2023 were testing interventions on patients with long COVID symptoms (retrieved from PubMed with the search terms “long-covid” AND “neuro”). All the seven RCTs excluded people with neurological and/or psychiatric conditions, people with moderate to severe cognitive impairment, or people with a disability, leaving an evidence gap regarding the potential benefits and risks of the interventions in these populations.

Was the extra burden and costs of enrolling people with cognitive impairment the hidden reason for their exclusion? Or was it because including them would have increased the sample heterogeneity generating noise that would have made the signal (treatment efficacy) more difficult to observe?

Whatever the reason, those meta-analyses and clinical trials cannot support policy or clinical decision-making on long COVID management for cognitively vulnerable patients (CVPs). The overlook or exclusion of CVPs from long COVID research not only reinforces the stigmatization of CVPs by disregarding cognitive disorders in research and health policy planning but also limits the generalization of findings on long COVID. Given the high prevalence of cognitive disorders in the population, the global prevalence of 6.2% of long COVID (1) may be an underestimation of the actual proportion of individuals suffering from long-term sequelae post-COVID-19 infection.

Witham and colleagues (4) provided a framework for addressing key issues when designing and delivering inclusive COVID-19 research, notably including strong community engagement, with exclusion criteria kept to a minimum. The literature on patient and public engagement is now abundant, and methodological tools and guidelines to include and account for a diversity of participants in research studies, even with cognitive disorders (3), can easily be found, leaving little excuse for their quasi-systematic exclusion.

However, there is another major issue in long COVID research that impedes the investigation of cognitive sequelae in people with cognitive disorders. The confounding nature of their symptomatology with the neurocognitive sequelae of severe acute respiratory syndrome coronavirus 2 (SARS-CoV-2) infection is an added layer of complexity for their inclusion in long COVID research and long COVID care.

The World Health Organization defines clinical cases of the post-COVID-19 condition as individuals that have “*new-onset or persisting symptoms 3 months after onset of symptomatic SARS-CoV-2 infection that were not pre-existing*” (5).

Given this definition and given that most, if not all, of their cognitive symptoms were already present before the COVID-19 infection in my fellow partners, their complaints are considered an exacerbation of their primary diagnoses’ symptoms, whatever the reasons for this exacerbation. That their symptoms worsened after the infection matters little in their access to long COVID care and cognitive rehabilitation.

In research, even if they could be included as participants, they might not meet the eligibility criteria of the test group depending on the long COVID case definition used and be misassigned in the control group, potentially leading to insignificant results regarding their risk of long COVID cognitive sequelae.

Obviously, to avoid confounded conclusions because of background noises, a careful analysis is required to identify whether post-acute COVID-19 sequelae are attributable and specific to SARS-CoV-2 infection, or whether they are driven by non-specific aspects of acute illness or the environment, or whether they are just similar in intensity or severity to symptoms pre-SAR-CoV-2 infection. However, as proposed by Shankar and colleagues for people with intellectual disabilities (6), any worsening of a cognitive symptom post-COVID-19 infection requiring a change in treatment or modifying daily functioning should be investigated as potential long COVID sequelae.

Indeed, including participants with neurologic and psychiatric disorders and broadening their case definition to any worsening symptoms post-infection, a few studies found that patients with neurocognitive involvement post-COVID-19 are more likely to have underlying cognitive or neurological disorders compared with those without (7–9), although no studies provided details on the pre-existing conditions.

In studies focused on the interplay of specific cognitive disorders with COVID-19, neurodegenerative dementia is the most actively studied, with parallels drawn from neuropathology to animal models (10, 11). Recently, a detailed cognitive and neuroimaging evaluation of patients with pre-existing dementia, 1 year following SARS-CoV-2 infection, showed a worsening of cognitive symptoms with a specific pattern of decreased attention, executive dysfunctions, delayed information processing speed, mood changes, and memory impairment, indicating an underlying disruption of frontal subcortical networks (12).

Conversely, the interplay between neurodevelopmental disorders and long-term COVID-19 has seldom received attention. Some studies reported an increased risk of long COVID in specific neurodevelopmental conditions or their associated traits, such as autism (13) and ADHD (14), but did not detail the types of long COVID symptoms associated with them. Although a report of a long COVID patient case with neurocognitive sequelae that had a pre-existing ADHD diagnosis suggested that fronto-executive network differences may form a selective vulnerability increasing the risk for cognitive symptoms (15), to my knowledge, no studies have specifically tested this hypothesis on this patient population nor on any neurodevelopmental disorder.

The main issues that prevent us from assessing the association between cognitive disorders and long COVID cognitive sequelae are combined in a recruiting RCT at McMaster University (Ontario, Canada), testing a cognitive rehabilitation intervention in patients with post-COVID neurocognitive sequelae (clinicaltrials.gov - NCT05676047). Although this represents a first-line treatment opportunity for my patient partners, they cannot benefit from it because of their “*previous history of a diagnosis of a neurological disorder affecting thinking*” (*Noise #1 (Background noise): Avoidance of confounding factors*). Some of them have no “*access to an electronic device with internet access and capability for Zoom videoconferencing*” (*Noise # 2 (Low sampling rate): Lack of inclusiveness*). Moreover, for those who would “pass” the two previous eligibility criteria, it is not said how their cognitive symptoms will be attributed “*to COVID and not to other intervening diagnoses associated with cognitive dysfunction*” (*Noise # 3 (Low signal quality): Lack of clarity on case definition*).

In 2024, and in light of the progress made to do inclusive research, can’t we do better than this for this already highly vulnerable group? “*Noise is part of the real world*” (16), and so are people with cognitive disorders. Instead of considering them as noises, let's start hearing their voices and improve long COVID research for a better care for all.

## Author contributions

CJ: Writing – original draft, Writing – review & editing.
